# Osmotic Demyelination Syndrome and Pituitary Apoplexy Following mRNA COVID-19 Vaccination: A Case Report

**DOI:** 10.3390/reports9020141

**Published:** 2026-04-30

**Authors:** Stylianos Kopanos, Ulrich J. Knappe, Nasreddin Abolmaali, Joachim Feldkamp

**Affiliations:** 1 Academic Department of Endocrinology, Diabetes and Infectiology, Klinikum Bielefeld, Medical School, University Medical Centre East Westphalia-Lippe, Bielefeld University, 33604 Bielefeld, Germany; joachim.feldkamp@klinikumbielefeld.de; 2 Department of Neurosurgery, Johannes Wesling Klinikum Minden, Medical Centre Ruhr University Bochum, 32429 Minden, Germany; ulrichjohannes.knappe@muehlenkreiskliniken.de; 3 Department of Diagnostic and Interventional Radiology, Klinikum Bielefeld, Medical School, University Medical Centre East Westphalia-Lippe, Bielefeld University, 33604 Bielefeld, Germany; nasreddin.abolmaali@klinikumbielefeld.de

**Keywords:** osmotic demyelination syndrome, hyponatremia, SIAD, pituitary apoplexy, hypopituitarism, mRNA vaccine

## Abstract

**Background and Clinical Significance**: Osmotic demyelination syndrome (ODS) and pituitary apoplexy are rare but potentially severe neurological and endocrine complications that can arise in the context of profound metabolic stress. **Case Presentation**: We describe the case of a previously healthy 34-year-old man who developed severe symptomatic hyponatremia shortly after receiving his second dose of an mRNA COVID-19 vaccine. Initial laboratory findings and clinical assessment were consistent with syndrome of inappropriate antidiuretic hormone secretion. Following correction of serum sodium, the patient experienced neurological deterioration with gait disturbance, dysarthria, and cognitive impairment. Follow-up brain MRI demonstrated extrapontine osmotic demyelination involving the basal ganglia and thalamus, despite initially normal imaging. During subsequent endocrinological follow-up, pituitary MRI revealed pituitary apoplexy in a previously unrecognized adenoma, accompanied by evolving partial hypopituitarism. The patient was managed with careful electrolyte control and long-term hormone replacement therapy, including hydrocortisone, levothyroxine, and recombinant growth hormone, resulting in gradual functional and cognitive improvement. **Conclusions**: This case highlights the interaction between severe hyponatremia, osmotic stress, and pituitary vulnerability, and emphasizes the need for cautious sodium correction, careful interpretation of temporal associations, and continued clinical vigilance in the context of COVID-19 vaccination programs.

## 1. Introduction and Clinical Significance 

Since the introduction of mRNA-based COVID-19 vaccines in 2020, millions of individuals have been immunized worldwide, with an overall favourable safety profile. The vaccines developed by Moderna and Pfizer-BioNTech have demonstrated high efficacy, and only rarely been associated with severe adverse events. Nevertheless, a growing number of case reports have described neurological and endocrine complications temporally related to vaccination, suggesting possible immune-mediated mechanisms in predisposed individuals [1].

Osmotic demyelination syndrome (ODS), formerly known as central pontine myelinolysis, is a rare but severe neurological condition characterized by non-inflammatory demyelination, typically affecting the pons and extrapontine structures such as the basal ganglia and thalamus [2]. It is classically associated with rapid correction of chronic hyponatremia, but may also occur in the context of metabolic or systemic stress, liver disease, or malnutrition. The pathogenesis involves osmotic shifts leading to cellular dehydration and injury of oligodendrocytes, resulting in demyelination. Clinical manifestations include dysarthria, dysphagia, paraparesis, and cognitive or behavioural changes [3].

The syndrome of inappropriate antidiuretic secretion (SIAD) represents one of the most frequent causes of euvolemic hyponatremia [4]. Several reports have described SIADH following mRNA COVID-19 vaccination, suggesting a transient inflammatory activation of hypothalamic or pituitary regulation of vasopressin release [5].

Pituitary apoplexy is another rare but potentially life-threatening condition resulting from haemorrhage or infarction within a pre-existing pituitary adenoma. Clinical features include acute headache, visual impairment, ophthalmoplegia, and hypopituitarism [6]. In recent literature, isolated cases of pituitary inflammation (hypophysitis) and apoplexy have been reported in temporal association with COVID-19 vaccination, often in individuals with undiagnosed adenomas. The underlying mechanism is thought to involve immune-mediated vasculitis, thrombogenic response, or local inflammatory stress on pituitary tissue [7].

The present report describes a unique and complex case of osmotic demyelination syndrome with extrapontine distribution and subsequent pituitary apoplexy occurring after Moderna mRNA vaccination in a previously healthy 34-year-old man. The case highlights the possible interplay between severe hyponatremia, immune activation, and pituitary vulnerability, emphasizing the need for vigilance in post-vaccination neurological or endocrine manifestations.

This report adheres to the CARE case report guidelines and includes a patient timeline and proposed mechanistic figure for transparency and reproducibility [8].

## 2. Case Presentation

A 34-year-old man with no significant past medical history received his first dose of the Moderna mRNA COVID-19 vaccine on 13 May 2021 without complications. After the second dose on 12 June 2021, he developed severe cephalgia within two hours. This persisted for seven to eight days, and was accompanied by general deterioration, hyperemesis, concentration difficulties, and disorientation. Electrocardiography revealed sinus rhythm and a Brugada type I pattern in lead V1. Clinical examination showed no signs of hypovolemia, including stable blood pressure and absence of orthostatic hypotension.

The patient had a normal body mass index, was not taking regular medications or supplements, and had no history of alcohol misuse, malnutrition, or prior electrolyte disturbances. SARS-CoV-2 infection was excluded by negative PCR testing.

After hospital admittance, laboratory findings demonstrated marked hyponatremia with sodium of 100 mmol/L (normal range: 135–145 mmol/L). Serum sodium increased from 100 to 119 mmol/L within 24 h, corresponding to a correction rate of 19 mmol/L, exceeding recommended limits. Brain MRI showed no FLAIR signal abnormalities, no demyelinating lesions or oedema, and no ventricular enlargement. The initial MRI did not demonstrate a clearly identifiable pituitary adenoma. Gastro- and colonoscopy demonstrated reflux esophagitis. The patient was discharged after seven days.

Two days after discharge, he was readmitted with new neurological symptoms, including hypomimia, gait unsteadiness, dysarthria, cognitive deterioration, mild aphasia, soft and slowed speech, and dysphagia. Cerebrospinal fluid analysis showed no intrathecal immunoglobulin synthesis, negative antineuronal antibodies, and normal cell count and protein. EEG was unremarkable. Serum sodium was again low, at 113 mmol/L.

**Figure 1:**
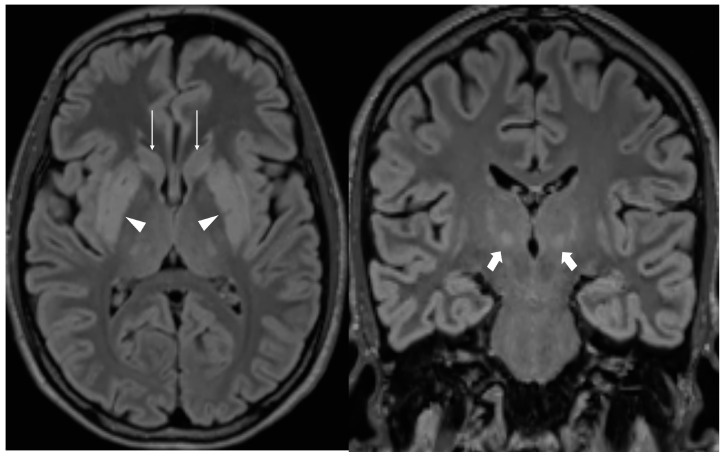
Coronal (**left**) and axial (**right**) FLAIR MRI sequences demonstrating bilateral hyperintense lesions in the putamen (arrowheads), caudate nucleus head (arrows), and thalamus (bold arrows) consistent with extrapontine osmotic demyelination syndrome.

Repeat MRI demonstrated bilateral hyperintense lesions in the putamen, caudate nucleus, thalamus, and pons, consistent with osmotic demyelination syndrome with extrapontine distribution (Figure 1). No significant compression of the optic chiasm was observed. Clinically, no extrapyramidal movement disorders were observed. No major diagnostic limitations—such as restricted access to imaging or laboratory testing—were encountered.

The patient received intravenous immunoglobulins (total dose 150 g over five days) based on an initial working hypothesis of a possible immune-mediated neurological process, given the acute neurological deterioration and the absence of a definitive diagnosis at that time. He was transferred to an intermediate care unit for controlled sodium substitution with 3% NaCl. Serum osmolality was 273 mmol/kg (normal range: 275–295 mmol/kg), urine osmolality 729 mmol/kg (normal range: 50–1200 mmol/kg) prior to fluid administration, and copeptin 3.9 pmol/L (normal range: <11.25 pmol/L). Therapy included fluid restriction and tolvaptan 3.75 mg daily.

During hospitalization, bicytopenia developed, with leukopenia of 3.4 × 10^9^/L (normal range: 4.0–10.0 × 10^9^/L) and haemoglobin of 11.5 g/dL (normal range: 13.5–17.5 g/dL), most likely secondary to immunoglobulin therapy. Transferrin saturation was 18% (normal range: 20–50%), and folate deficiency was detected. Bone marrow biopsy and CT imaging excluded malignancy. Neuron-specific enolase (NSE) was 23.5 ng/mL (normal range: <16.3 ng/mL).

Incidental laboratory findings revealed hypocortisolism with a morning cortisol of 24 µg/L (normal range: 50–250 µg/L) and ACTH of 17 pg/mL (normal range: 10–60 pg/mL). An ACTH stimulation test showed an adequate cortisol rise. No exogenous glucocorticoid therapy was administered prior to endocrine assessment. Sodium normalized to 142 mmol/L at discharge.

**Figure 2:**
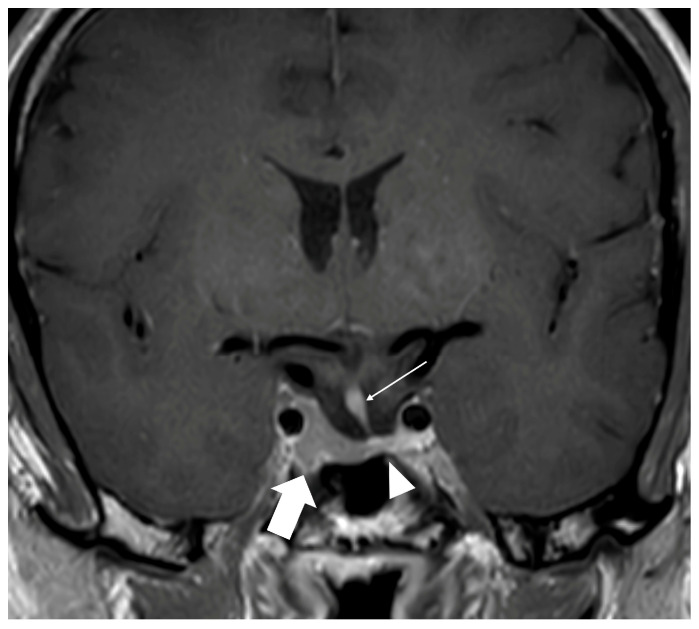
Coronal MRI section showing pituitary left-sided apoplexy (arrowhead) with right-sided enlargement (bold arrow), partial surrounding of the carotid artery (Knosp grade II) and deviation of the pituitary stalk (arrow).

Three months later, the patient was referred to our clinic for the first time; this was because partial adrenocorticotropic insufficiency was suspected by the general practitioner. Laboratory evaluation as seen in Table 1 confirmed secondary hypocortisolism with inappropriately normal ACTH of 10.6 pg/mL (normal range: 5–55), cortisol 1.83 µg/L (normal range: 5–25 µg/L), and impaired thyrotropic and somatotropic axes (inadequate low TSH 2.6 mIU/L, normal range: 0.4–4.0; free T3 2.6 pg/mL, normal range: 2.0–4.4; free T4 0.7 ng/dL, normal range: 0.8–1.8; IGF-1 62 ng/mL, normal range: 88–246). Serum sodium remained within the normal range during follow-up. At the time of endocrine evaluation, the patient was not receiving regular medication. Pituitary MRI demonstrated a pituitary apoplexy with deviation of the gland to the left and signs of a right sided residual pituitary adenoma (Knosp grade 2) (Figure 2). Surgical intervention was not indicated, as there were no visual deficits or signs of mass effect.

The patient reported fatigue, concentration and word-finding difficulties, an increased sleep requirement, and overall reduced physical and cognitive performance. Hydrocortisone 5-5-0 mg/day and L-thyroxine 50 µg/day were initiated. A relatively low initial hydrocortisone dose was selected due to the mild clinical presentation; this was subsequently adjusted based on clinical response. Because of normal gonadotropin levels and mid-normal testosterone without clinical signs of hypogonadism, such as reduced libido, testosterone replacement therapy was not necessary.

Combined dynamic endocrine testing later confirmed insufficient ACTH and cortisol response, and reduced IGF-1 levels, while gonadotropins, testosterone, and prolactin remained within normal ranges, indicating partial hypopituitarism. Growth hormone deficiency was supported by reduced IGF-1 levels and confirmed by dynamic endocrine testing.

Over the following 3 years, the patient continued to experience reduced stamina and productivity, as well as diminished mental and physical fitness. Repeat testing confirmed persistent insufficiency. Recombinant human growth hormone (0.2 mg subcutaneously daily) was introduced together with L-thyroxine dose adjustment, leading to marked clinical improvement. The patient reported no nocturia and only mild thirst, without indication for desmopressin therapy. Due to persistent midday fatigue, hydrocortisone was increased to 10-5-0 mg/day, but this was later reduced again as no clinical benefit was reported by the patient.

Psychological tests revealed significant deficits in sustained attention and alertness (percentile rank 8–10), while flexibility and adaptability remained within the normal range. The hypothalamic–pituitary–adrenal axis and the hypothalamic–pituitary–thyroid axis is still under evaluation on regular follow-up in our outpatient clinic.

## 3. Discussion

**Figure 3:**
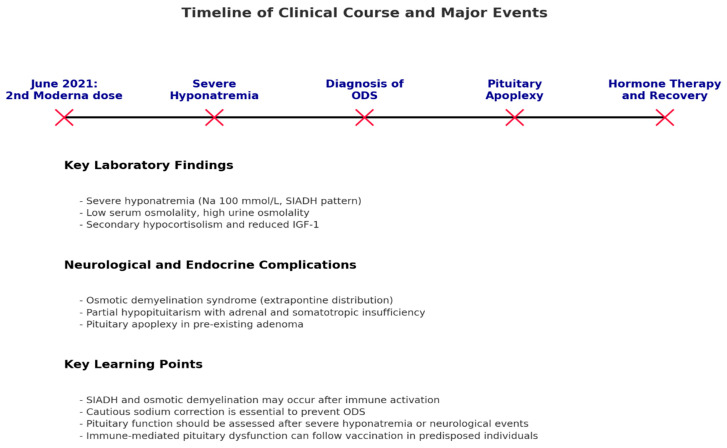
Timeline of the clinical course showing temporal sequence from mRNA vaccination to diagnosis, treatment, and follow-up. Rapid sodium correction (>10 mmol/L/24 h) contributed to the development of osmotic demyelination syndrome. ODS: Osmotic Demyelination Syndrome; SIADH: Syndrome of Inappropriate Antidiuretic Hormone Secretion.

This case illustrates a rare constellation of neurological and endocrine complications following mRNA-based COVID-19 vaccination, characterized by severe hyponatremia, osmotic demyelination syndrome (ODS), and subsequent partial hypopituitarism secondary to pituitary apoplexy. The clinical sequence, temporal relationship, and imaging findings suggest a multifactorial pathogenesis combining immunological, metabolic, and vascular mechanisms (Figure 3). Similar endocrine and neurological complications have been reported, albeit rarely, following other vaccinations.

Hyponatremia is a frequent electrolyte disturbance in hospitalized patients, but sodium levels below 110 mmol/L, as observed in this case (100 mmol/L), are uncommon and often life-threatening. The initial presentation of severe hyponatremia shortly after vaccination, associated with low serum osmolality (273 mmol/kg; normal range: 275–295 mmol/kg) and inappropriately high urine osmolality (729 mmol/kg), was consistent with the syndrome of inappropriate antidiuretic secretion (SIAD). SIAD after mRNA COVID-19 vaccination has been sporadically reported and may reflect transient hypothalamic or pituitary inflammation, cytokine-mediated arginine vasopressin (AVP) release, or an immune-induced alteration of osmotic regulation. Prolactin levels remained within the normal range, suggesting partial rather than complete stalk involvement.

Osmotic demyelination syndrome remains one of the most severe complications of sodium correction, classically linked to overly rapid correction of chronic hyponatremia. In this case, sodium was increased by 19 mmol/L within 24 h, a rate that exceeded the recommended limit of 8–10 mmol/L per day. MRI findings demonstrated bilateral signal abnormalities in the basal ganglia, thalamus, and pons, consistent with extrapontine ODS. Although correction speed was probably the key determinant factor, the patient’s systemic condition—characterized by dehydration, vomiting, and metabolic stress—may have increased cellular vulnerability to osmotic injury [9].

A clear temporal association was observed between mRNA vaccination and the onset of hyponatremia, osmotic demyelination, and pituitary dysfunction. The presumed sequence of events involved fluid loss with diarrhoea and general deterioration leading to hyponatremia, followed by osmotic demyelination. The osmotic demyelination was the potentially precipitating factor leading to subsequent pituitary apoplexy, possibly in the context of a pre-existing pituitary adenoma and immune activation after vaccination [10]. Differential diagnoses such as autoimmune encephalitis, acute adrenal crisis, myasthenia gravis, and ischemic or demyelinating disorders were excluded based on normal cerebrospinal fluid and EEG findings, an adequate ACTH stimulation response, and an MRI pattern inconsistent with inflammatory or vascular lesions.

**Figure 4:**
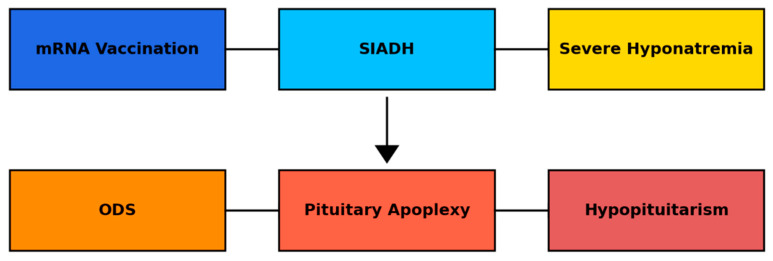
Proposed pathophysiological mechanism linking mRNA vaccination to SIADH, severe hyponatremia, ODS, pituitary apoplexy, and subsequent hypopituitarism. ODS: Osmotic Demyelination Syndrome; SIADH: Syndrome of Inappropriate Antidiuretic Hormone Secretion.

Interestingly, ODS in this case coincided with subsequent pituitary dysfunction, although the temporal coexistence of ODS and pituitary dysfunction does not imply a direct causal relationship. Pituitary apoplexy was demonstrated radiologically as haemorrhagic or ischemic infarction within a pre-existing adenoma. Several potential mechanisms could explain this association. First, ODS-related osmotic and microvascular stress may have affected the pituitary and hypothalamic region, predisposing to ischemic injury [11]. Although the clinical presentation occurred in temporal proximity to vaccination, rapid correction of profound hyponatremia remains the most established explanation for ODS in this case, and causality cannot be inferred. Second, repeated episodes of severe hyponatremia could have impaired pituitary perfusion and oxygen delivery, further promoting infarction (Figure 4).

While causality cannot be established, similar reports have described hypophysitis and apoplexy shortly after mRNA vaccination, particularly with Moderna or Pfizer vaccines. In most cases, the pathogenesis was presumed to be immune-mediated, possibly involving type IV hypersensitivity or molecular mimicry directed against pituitary antigens. The immune response may also have been amplified by local inflammation or pre-existing pituitary adenoma [12].

Endocrine evaluation during follow-up confirmed secondary adrenal and thyreotropic insufficiency and impaired somatotropic function, while gonadotropin and prolactin secretion remained intact. ACTH stimulation testing demonstrated an adequate cortisol response initially, while follow-up testing showed impaired response. Hypopituitarism patterns may vary, and can include panhypopituitarism in some cases. This partial pattern of hypopituitarism is typical of pituitary apoplexy, where selective vulnerability of corticotropes and somatotrophs is often observed. Replacement therapy with hydrocortisone and L-thyroxine resulted in partial symptomatic improvement, and subsequent introduction of recombinant growth hormone led to significant recovery of vitality and cognitive function. In addition to its endocrine role, insulin-like growth factor 1 (IGF-1) exerts important effects within the central nervous system, where it contributes to neuronal survival, synaptic plasticity, and cognitive function. Reduced IGF-1 levels have been associated with impaired neurocognitive performance and decreased neuronal resilience, which may have contributed to the persistent cognitive symptoms observed in this patient [13]. The absence of diabetes insipidus and preserved thirst regulation further supported an isolated anterior pituitary involvement. Even moderate overcorrection in susceptible patients can result in irreversible demyelination. Moreover, the coexistence of ODS and pituitary apoplexy raises awareness that the hypothalamic–pituitary axis can be secondarily affected by osmotic or immune injury [14].

The pathophysiological link between mRNA vaccination and the observed complications remains speculative but biologically plausible. Transient cytokine release (interleukin-6, TNF-α), endothelial activation, and immune-mediated vasculitis have been proposed mechanisms in post-vaccine inflammatory events involving the central nervous system [15,16]. In this case, these factors may have contributed to both SIAD and pituitary ischemia, superimposed on osmotic vulnerability due to hyponatremia correction.

To our knowledge, this represents one of the few reported cases of osmotic demyelination syndrome with extrapontine involvement following a too-rapid correction of sodium and subsequent pituitary apoplexy following mRNA COVID-19 vaccination. It highlights the complex interaction between immune activation, electrolyte disturbances, and pituitary vulnerability. Clinicians should remain alert to neurological or endocrine symptoms following vaccination, especially when accompanied by unexplained hyponatremia or altered consciousness.

## 4. Conclusions

This case highlights a rare and complex neuroendocrine presentation occurring in temporal proximity to mRNA COVID-19 vaccination. Rapid correction of severe hyponatremia represents the most established mechanism underlying osmotic demyelination syndrome in this patient, while pituitary apoplexy likely reflects secondary vulnerability to metabolic and vascular stress. Although causality cannot be proven, careful interpretation of temporal associations, cautious sodium correction, and long-term endocrine follow-up are essential in similar clinical scenarios.

## Figures and Tables

**Table 1:** Summary of endocrine findings.

Parameter	Value	Reference Range	Interpretation
Cortisol (morning)	1.83 µg/dL	5–25 µg/L	Low
ACTH	10.6 pg/mL	5–55 pg/mL	Inappropriately normal
ACTH stimulation test (initial)	Adequate response	—	Preserved adrenal reserve initially
ACTH stimulation test (follow-up)	Impaired response	—	Secondary adrenal insufficiency
TSH	2.6 mIU/L	0.4–4.0 mIU/L	Inappropriately normal
Free T4	0.7 ng/dL	0.8–1.8 ng/dL	Low
Free T3	2.6 pg/mL	2.0–4.4 pg/mL	Low–normal
IGF-1	62 ng/mL	88–246 ng/mL	Low
Gonadotropins (LH/FSH)	Within normal range	—	Preserved
Testosterone	Within normal range	—	Preserved
Prolactin	Within normal range	—	Preserved

## Data Availability

Original data generated and analysed during this study are included in this published article.

## Abbreviations

The following abbreviations are used in this manuscript:

ODS	Osmotic Demyelination Syndrome
SIAD	Syndrome of Inappropriate Antidiuresis
MRI	Magnetic Resonance Imaging
ACTH	Adrenocorticotropic Hormone
IGF-1	Insulin-like growth factor 1
